# Spinal Changes of a Newly Isolated Neuropeptide Endomorphin-2 Concomitant with Vincristine-Induced Allodynia

**DOI:** 10.1371/journal.pone.0089583

**Published:** 2014-02-24

**Authors:** Yang Yang, Yong-Gang Zhang, Guo-An Lin, He-Qiu Xie, Hai-Tao Pan, Ben-Qing Huang, Ji-Dong Liu, Hui Liu, Nan Zhang, Li Li, Jian-Hua Chen

**Affiliations:** 1 Department of Neurosurgery, The Zhumadian City Center Hospital, Zhumadian, P.R. China; 2 Department of ICU, The Zhumadian City Center Hospital, Zhumadian, P.R. China; 3 Department of Burn, The 159th Hospital of PLA, Zhumadian, P.R. China; 4 Department of Pain, The Zhumadian City Center Hospital, Zhumadian, P.R. China; 5 School of Statistics, Xi’an University of Finance and Economics, Xi’an, P.R. China; 6 Department of Oncology, The 159th Hospital of PLA, Zhumadian, P.R. China; 7 Department of student affairs, Xinxiang Medical University, Xinxiang, P.R. China; University of Texas Medical Branch, United States of America

## Abstract

Chemotherapy-induced neuropathic pain (CNP) is the major dose-limiting factor in cancer chemotherapy. However, the neural mechanisms underlying CNP remain unclear. There is increasing evidence implicating the involvement of spinal endomorphin-2 (EM2) in neuropathic pain. In this study, we used a vincristine-evoked rat CNP model displaying mechanical allodynia and central sensitization, and observed a significant decrease in the expression of spinal EM2 in CNP. Also, while intrathecal administration of exogenous EM2 attenuated allodynia and central sensitization, the mu-opioid receptor antagonist β-funaltrexamine facilitated these events. We found that the reduction in spinal EM2 was mediated by increased activity of dipeptidylpeptidase IV, possibly as a consequence of chemotherapy-induced oxidative stress. Taken together, our findings suggest that a decrease in spinal EM2 expression causes the loss of endogenous analgesia and leads to enhanced pain sensation in CNP.

## Introduction

Chemotherapy-induced neuropathic pain (CNP) is a common, dose-limiting side effect of cancer treatment using chemotherapeutic drugs. CNP limits the maximum dose of drug that can be safely administered as well as the duration of treatment, and hence impairs the quality of life [1]. Unlike neuropathic pain induced by trauma and diabetes, CNP occurs in the absence of axonal degeneration in peripheral nerves, suggesting that the mechanisms underlying CNP are elusive and complex [2], [3]. CNP is often resistant to standard analgesics. Hence, it is crucial to investigate the mechanisms causing CNP and subsequently devise effective treatment strategies [4].

Opioid receptors in the spinal cord play a critical role in modulating nociceptive transmission. Approximately 70% of opiate ligand-mediated signaling in the spinal cord occurs through the mu-opioid receptor (MOR); the most potent opiate drugs are known to act as ligands of MOR [5]. Endomorphin-1 (EM1) and endomorphin-2 (EM2) were newly isolated endogenous opioid peptides and identified as the endogenous ligands of MOR [6]–[8]. While EM1-like immunoreactivity (-LI) is primarily restricted to the brain, EM2-LI is found mainly in the spinal cord and suspected to modulate pain signaling at that level [9], [10]. In rat models of neuropathic pain, administration of exogenous EM2 at the spinal level results in a much stronger analgesic effect than morphine [11]–[13]. Following partial ligation of the sciatic nerve, EM2-LI in the spinal dorsal horn ipsilateral to the nerve injury was shown to be greatly reduced, suggesting that the loss of endogenous inhibitory signals might be responsible for the subsequent chronic pain [14].

One study reported that paclitaxel- and vincristine-induced CNP seems relatively resistant to opioid therapy, and that large doses of morphine alone did not have a significant analgesic effect [15]. While studying the relationship between the spinal opioid system and CNP, two recent studies demonstrated the involvement of spinal opioid receptors in electroacupuncture or magnetic stimulation induced analgesia in CNP. They showed that administration of opioid receptor antagonists could successfully block electroacupuncture or magnetic stimulation mediated inhibition of allodynia and hyperalgesia in rat CNP models [16], [17]. To further understand the pathophysiology of CNP, it is essential to determine whether the expression of endogenous inhibitory peptides such as EM1 and EM2 is modified in CNP. No studies thus far have investigated the relationship between spinal EM2 and CNP.

In this study, we examined the role of spinal EM2 in the pathophysiology of CNP. We used a rat model of CNP implanted with a mini-osmotic pump to continuously deliver vincristine sulfate, and analyzed changes in the expression of EM2 in the spinal cord during the development and progression of CNP. Further, we examined the threshold of pain tolerance following intrathecal administration of β-funaltrexamine (β-FNA) and exogenous EM2. In addition, to determine whether the reduction in spinal EM2 expression is a consequence of increased activity of dipeptidylpeptidase IV (DPP IV), we treated our CNP models systemically with diprotin A, an inhibitor of DPP IV. We also investigated the role of chemotherapy- induced oxidative stress in modulating the activity of DPP IV.

## Materials and Methods

### Animals

Adult male Sprague–Dawley rats, weighing 200 g, were used. Rats were housed under standard conditions. All procedures of our experiments were approved by the Committee of Animal Use for Research and Education of Zhumadian City Center Hospital (Zhumadian, Henan Province, P.R. China), and all efforts were made to minimize the number of animals used and their suffering [18]. (Permit Number: zmd-13-6688).

### Mini-osmotic Pump Implantation

As described previously [19], [20], rats were anesthetized with halothane (5% to induce, 2–3% to maintain), and their right external jugular vein was catheterized with a vincristine-filled miniosmotic pump (0.5 µl/h, 14 days; Alzet Model 2002, Durect Corporation, Cupertino, CA, USA) that had been primed overnight to deliver 30 mg/kg/day vincristine sulfate (Sigma-Aldrich, St Louis, MO, USA) (Vincristine group). Control rats were implanted with primed mini-osmotic pump containing 0.9% saline (Sham group).

### Drugs

Endomorphin-2, Endomorphin-1, β-funaltrexamine (β-FNA, antagonist for mu-opioid receptor), phenyl N-tert-butylnitrone (PBN, scavenger for reactive oxygen species) and diprotin A (inhibitor of dipeptidylpeptidase IV) were purchased from Sigma-Aldrich.

### Experimental Design

In the first series of experiments, rats were divided into Naive group, Sham group, Vincristine group. The baseline values of pain behavior were recorded before osmotic pump implantation. Then, from 1 day post osmotic pump implantation (1d) to 28 days post osmotic pump implantation (28d), pain behavioral tests were performed once a day in each group (n = 10/group). Body weight and motor function were detected once a week to monitor the toxicity of vincristine (n = 10/group).

In the second series of experiments, rats in these three groups were sacrificed on 3d, 5d, 7d, 10d, 14d and 21d, and the spinal cords and dorsal root ganglia were harvested and preserved (n = 5/d in Naive and Sham groups, n = 10/d in Vincristine group). The spinal cords were used for immunostaining of EM2, EM1 or MOR, Western blot analysis of MOR, the detection of spinal content of EM2 or EM1, spinal activity of dipeptidylpeptidase IV and spinal reactive oxygen species; the dorsal root ganglia were used for immunostaining of EM2 and the detection of the content of EM2.

In the third series of experiments, rats of Vincristine group were injected intraperitoneally with PBN (1.5 mg/kg/d) [21], [22] from 1d to 7d. The spinal cords of rats were harvested for detection of activity of DPP IV after pain behavioral test on 3d, 5d, 7d, 10d, 14d and 21d (n = 10/d).

In the fourth series of experiments, rats of Vincristine group were injected intraperitoneally with diprotin A (3.5 mg/kg/d) [23], [24] from 1d to 7d. The spinal cords of rats were harvested for detection of content of EM2 after pain behavioral test on 3d, 5d, 7d, 10d, 14d and 21d (n = 10/d).

In the fifth series of experiments, saline, EM2, EM1 or β-FNA was injected intrathecally in Vincristine group at 2 weeks post osmotic pump implantation (2w). After injection, pain behavior was immediately measured (n = 10).

In the sixth series of experiments, saline, EM2 or β-FNA was injected intrathecally in Vincristine group at 2w, and a total of 30 wide dynamic range (WDR) neurons were immediately recorded in each group (Sham group, Vincristine+Saline group, Vincristine+EM2 group and Vincristine+β-FNA group; n = 30/group).

### Rotarod Test

In order to assess whether the dosage of vincristine used in the present experiment could impair motor function, which might influence the pain behavioral results, the motor function of Vincristine group was assessed by the rotarod test [25]. Rats were placed on the 7650 Rotarod accelerator treadmill (Ugo Basile, Varese, Italy) set at a constant speed of 25 RPM. As the animal took a grip of the drum, the accelerator mode was selected on the treadmill, the rotation rate of the drum was increased linearly. Thereafter, the time was measured from the start of the acceleration period until the rat fell off the drum. The cut-off time was 30 s.

### EM Content Assay

EM2 or EM1 content was measured from spinal cord or dorsal root ganglion using a commercially available enzyme-linked immunosorbent assay kit (S12460001, Bachem, King of Prussia, PA, USA) [26]. The rats were euthanized and the L4-5 spinal cords or dorsal root ganglia were removed and immediately frozen in liquid nitrogen. Tissue samples were sonicated in 200 µl of lysis buffer, centrifuged at 17,000 *g* for 15 min, and the supernatant collected. In the assay, tissue EM2 or EM1 competed with exogenously added biotinylated EM2 or EM1 for a limited amount of immobilized antibody binding sites and the amount of biotinylated EM2 or EM1 was determined by colorimetric analysis. The amount of EM2 or EM1 present in the tissue samples was calculated from standard curve of known EM2 or EM1 concentration and was expressed in ng/mg protein. A portion of the tissue supernatant was used to measure total protein using a standard Bradford assay (BioRad, Hercules, CA, USA).

### Spinal Reactive Oxygen Species (ROS) Assay

The rats were euthanized and the spinal cord was dissected and homogenized. The supernatants were collected for ROS assay. The production of ROS was measured by 2′,7′-Dichlorodihydrofluorescein (DCFH) oxidation as described previously [27]. DCFH reacts with ROS to form 2′,7′-dichlorofluorescein (DCF), the fluorescent product. The fluorescence was read at 485 nm excitation and 530 nm emission on a fluorescence plate reader. The results were calculated as percentage of Vincristine 14d group.

### Measurement of Spinal Dipeptidylpeptidase IV (DPP IV) Activity

The rats were euthanized and the spinal cord was dissected and homogenized. The supernatants were collected for DPP IV activity assay. DPP IV activity of the spinal cord was measured by using the chromogenic substrate Gly-Pro-p-nitroanilide (Gly-Pro-pNA; Sigma) as previously reported [24]. 100 µl of supernatant was incubated at 37°C with 1.5 mM Gly-Pro-pNA in 100 µl of phosphate-buffered saline containing 10 mg/ml bovine serum albumin. Absorbance was measured at 405 nm on a microplate reader at 3-min intervals for a total of 60 min, and the number of picomoles of pNA formed was calculated by comparison to a pNA standard curve. The results were plotted as picomoles of pNA versus time, giving a measure of DPP IV activity expressed as picomoles per minute. The peak pNA production rate was used for analysis between groups.

### Intrathecal Catheter Insertion and Drug Administration

A polyethylene-10 catheter (Becton-Dickinson, Sparks, MD, USA) was intrathecally inserted according to a previous method [28]. The rats were allowed to recover for 1 day. Only the rats judged as neurologically normal were used for the subsequent drug administration. The dosage of each drug used in the present study was according to the previous reports. Treatment group received intrathecal injection of EM2 (0.3, 1, 3 µg) [13] or EM1 (3 µg) [13] or β-FNA (3, 10 µg) [29], while the same volume of normal saline was injected in control group. Each drug was dissolved in 10 µl of saline and injected intrathecally by the way of a single acute administration.

### Pain Behavioral Test

For the pain behavioral tests, the same room in which the rats were routinely housed was used. The rats were placed under inverted plastic boxes (30×30×50 cm^3^) on an elevated mesh floor and allowed to habituate for at least 15 min before the threshold testing. The observers of the behaviors were blinded to the treatment of the rats. To observe how different drug treatments affected the pain threshold, behavioral tests were performed 6 h before the drugs administration to provide baseline scores. After intrathecal drug injection, pain threshold was measured for every 10 min.

#### Von frey monofilaments

As previously described [30], a set of von Frey monofilaments (Stoelting, Chicago, IL, USA) was used to test the mechanical withdrawal threshold of the hindpaws. The monofilaments were applied with increasing force until the rat withdrew the paw. Lifting of the paw due to normal locomotory behavior was ignored. Each monofilament was applied five times. The threshold was taken as the lowest force that evoked a brisk withdrawal response to one of the five repetitive stimuli. The withdrawal thresholds were measured three or four times in order to obtain two consecutive values that differed not more than 10%.

#### Hargreaves’ method

Thermal nociceptive threshold to radiant heat was quantified using the paw withdrawal test [31]. Briefly, rats were placed in a Perspex enclosure, without restraint and a movable infrared radiant heat source placed directly under the plantar surface of the hind paw (Ugo Basile, Italy). The paw withdrawal latency to radiant heat was defined as the time from onset of the radiant heat to the withdrawal of the rat hind paw. The average of three estimations was taken to yield a mean paw withdrawal latency.

### Electrophysiological Testing

The rats were anesthetized and artificially ventilated. Core body temperature was monitored and maintained at 37.5±0.5°C. A laminectomy was performed from the T13 to L2 vertebrae to expose the lumbosacral enlargement of the spinal cord. Extracellular single unit recordings were made from L4-5 spinal dorsal horn with glass capillary microelectrodes (10–15 MΩ filled with 0.5 M sodium acetate). According to previous reports [32], [33], the dorsal horn neurons were identified as WDR units on the basis of their characteristic responses: (1) having a receptive field consisting of a small low threshold center and a large high threshold surround ipsilateral to the recording site; (2) responding with an increasing firing rate to brush, pressure and noxious pinch applied to the low threshold center; (3) showing no apparent accommodation when continuous noxious stimulation was applied. After successful identification of a single WDR unit, the unit responsiveness to 10 s mechanical stimuli was recorded. The spike trains were monitored with a memory oscilloscope and the numbers of neuronal firing were simultaneously recorded and saved on a computer via an A/D converter following spike discriminator and counter.

### Immunohistochemical Staining

#### Tissue preparation

Rats were anesthetized and perfused transcardially with paraformaldehyde. The L4-5 spinal cords and dorsal root ganglia were removed and transferred into 30% sucrose in 0.1 M phosphate buffer (PB, pH 7.4) for cryoprotection. The spinal cords were cut into 30 µm thick sections and collected in 0.01 M phosphate-buffered saline (PBS, pH 7.4). The dorsal root ganglia were cut into longitudinal sections measuring 10 µm thick and mounted onto gelatin-coated glass slides.

#### Immunofluorescence

After washed in PBS, the spinal cord sections were incubated at room temperature with rabbit antiserum against EM2 (AB5104, 1∶200; Chemicon, Temecula, CA, USA), rabbit antiserum against EM1 (AB5102, 1∶200; Chemicon) or guinea pig antiserum against MOR (AB1774, 1∶3000; Chemicon) in 0.0l M PBS containing 5% (v/v) normal donkey serum (NDS), 0.3% (v/v) Triton X-100, 0.05% (w/v) NaN3 and 0.25% (w/v) carrageenan (PBS-NDS, pH 7.4) for 24 h, followed by biotinylated goat anti-rabbit IgG (BA-1000, 1∶200; Vector Laboratories, Burlingame, CA, USA) or biotinylated goat anti-guinea pig IgG (BA-7000, 1∶200; Vector) diluted with PBS-NDS for another 4 h, and then with avidin-biotin-peroxidase complex Elite Kit (1∶100 dilution; Vector) in PBS for 1 h. Finally, the sections were reacted with 0.05 M Tris–HCl buffer (pH 7.6) containing 0.04% diaminobenzidine tetrahydrochloride (Dojin, Kumamoto, Japan) and 0.0003% H_2_O_2_ for visualizing EM2-LI, EM1-LI or MOR-LI. After staining, all the sections were observed with a bright-field microscope (Olympus AH2 VANOX, Tokyo, Japan). The dorsal root ganglion sections were incubated sequentially with: (1) rabbit antiserum against EM2 (1∶200) in PBS-NDS for 48 h at 4°C; (2) Cy3-labeled donkey anti-rabbit IgG (AP184F, 1∶200; Chemicon) in PBS-NDS for 12 h at 4°C. After staining, all the sections were observed with Olympus FV1000 confocal laser scanning microscope.

### Western Blot Analysis

Rats were anesthetized and the lumbar segments of spinal cord innervated by the L4-5 dorsal roots were rapidly removed. The collected tissue was mechanically homogenized and centrifuged. The supernatant was collected and stored at –80°C. Protein concentrations of the supernatant were determined using the BCA Protein Assay Kit (Pierce, Rockford, IL, USA). Proteins of interest were separated by SDS-PAGE electrophoresis (20 µg of total protein per well), and transferred onto nitrocellulose membranes. The membranes were placed in a blocking solution (Tris-buffered saline with 0.02% Tween and 5% non-fat dry milk powder) for 1 h, and incubated overnight with guinea pig antiserum against MOR (AB1774, 1∶1000; Chemicon). After washing, the membranes were incubated in peroxidase-conjugated secondary antibody (1∶1000; Santa Cruz Biotechnology, Santa Cruz, CA, USA) for 1 h, and then the membranes were detected by the enhanced chemiluminescence detection method (Amersham Pharmacia Biotech Inc., Piscataway, NJ, USA). The densities of protein blots were analyzed by using Labworks Software (Ultra-Violet Products Ltd., Cambridge, UK) and normalized to β-actin levels.

### Data Analysis

All data were collected by experimenters blinded to the surgery and reagents treatments and statistical analyses were done by using SPSS software (version 12). Data were expressed as mean ±standard error mean (mean±SEM). Statistical analysis of the data was carried out with a one-way analysis of variance (ANOVA) followed by Bonferroni post hoc analysis. A Pearson correlation was used to determine the correlation between the content of EM2 and pain behavioral performance. Comparisons between two means were performed by a Student’s T-test. Significance level was set at P<0.05.

## Results

### Mechanical Allodynia Occurred in Vincristine Treated Rats

**Figure 1:**
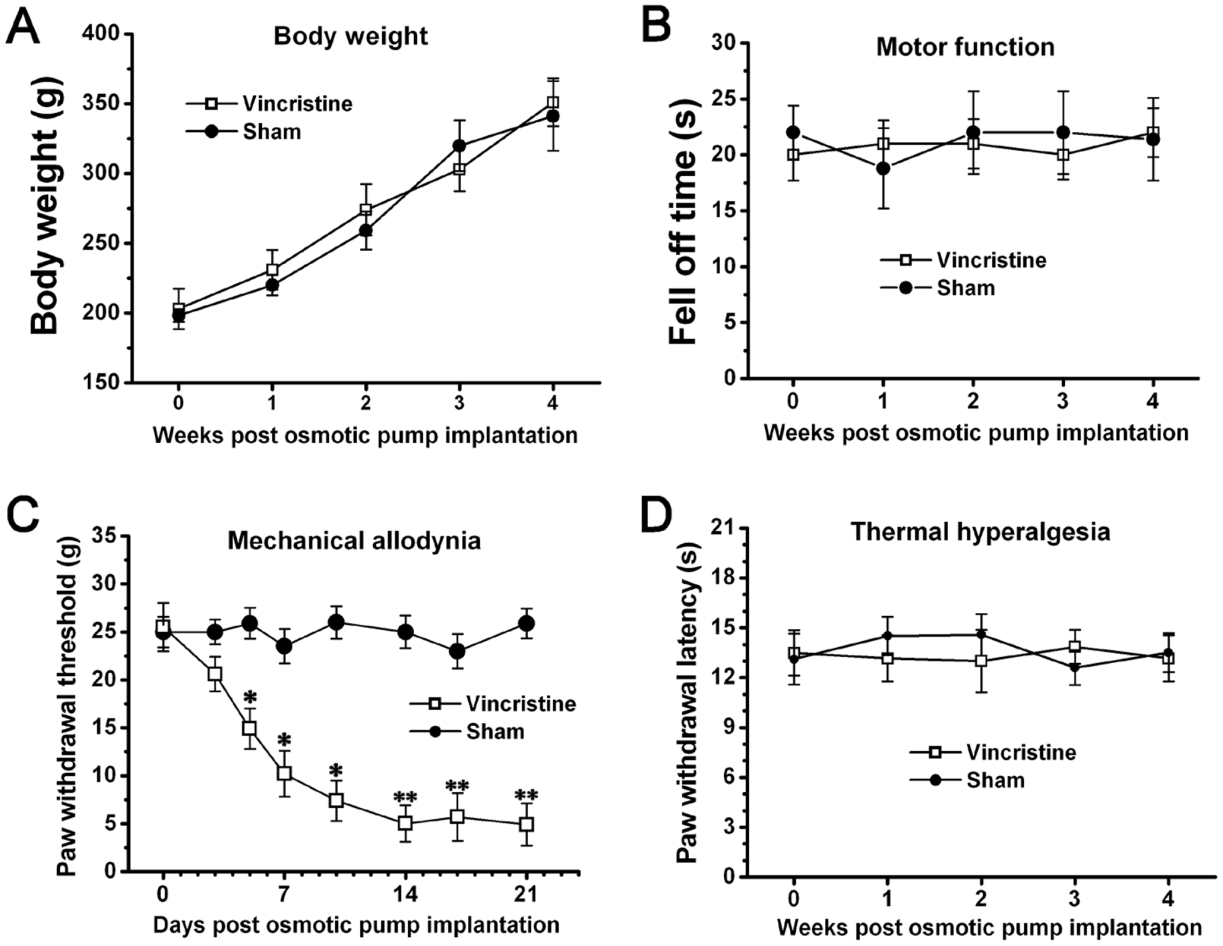
Mechanical allodynia occurred in vincristine treated rats. With regard to (A) body weight and (B) motor function, there was no difference between Vincristine group and Sham group. (C) Compared to Sham group, the paw withdrawal threshold of Vincristine group was significantly decreased. (D) No significant changes in thermal withdrawal thresholds were observed between Vincristine group and Sham group. All data were calculated as mean ± SEM (n = 10/group). *P<0.05, **P<0.01 vs. Sham group in C.

No difference in body weight was observed between Sham group (341±25.1 g; 4w) and Vincristine group (351±17.1 g; 4w), and the body weight of these two groups was persistently elevated through the period tested (Fig. 1A). Also, no difference in motor function was observed between Sham group (21.4±3.7 s; 4w) and Vincristine group (22±2.2 s; 4w), and these values were maintained at normal levels at all time points tested (Fig. 1B). These data indicated that vincristine treatment exerted no significant toxicity.

Compared to Sham group (25.9±1.6 g; 5d), the paw withdrawal threshold of Vincristine group was significantly decreased at 5d (14.9±2.1 g), reached the lowest value at 14d (5.3±1.4 g), and thereafter was at persistent low level (n = 10/group; P<0.05) (Fig. 1C). In contrast with the severe mechanical allodynia, no significant changes in thermal withdrawal thresholds were observed between Sham group (13.5±1.2 s; 4w) and Vincristine group (13.2±1.4 s; 4w) (Fig. 1D). These data indicated that vincristine treatment induced mechanical allodynia.

### Spinal EM2 was Significantly Decreased after Chemotherapy

**Figure 2:**
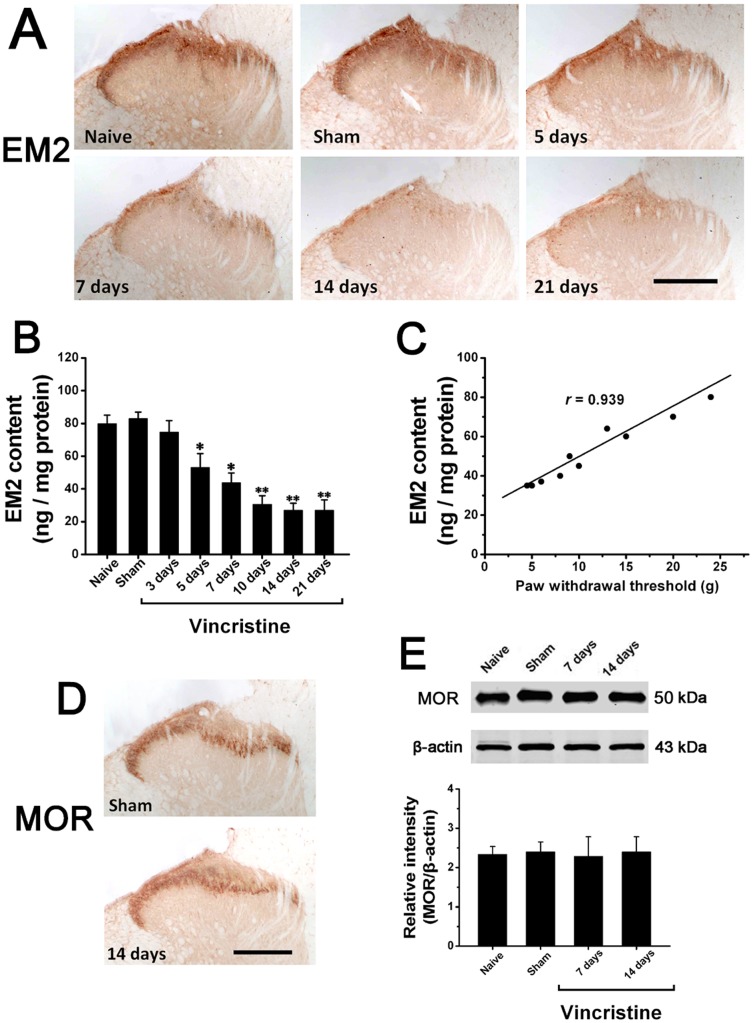
Spinal EM2 was significantly decreased in vincristine treated rats. (A) Compared to Sham group, EM2-like immunoreactivity in spinal dorsal horn of Vincristine group was significantly decreased. Bar = 200 µm. (B) Compared to Naive group and Sham group, ELISA showed that spinal EM2 content was significantly decreased in Vincristine group. (C) The content level of EM2 was found to be significantly correlated to the paw withdrawal threshold in Vincristine group (P<0.001, r = 0.939). (C) With regard to MOR-like immunoreactivity in spinal dorsal horn, there was no difference between Sham group and Vincristine group. Bar = 200 µm. (D) With regard to MOR expression in spinal cord, Western blot analysis showed that there was no difference between Sham group and Vincristine group. In vincristine treated rats, MOR expression was unchanged through the period tested. All data were calculated as mean ± SEM (n = 10/group). *P<0.05, **P<0.01 vs. Naive group and Sham group in (B). EM2: endomorphin2; MOR: mu-opioid receptor.

Immunohistochemistry indicated that compared to Naive group and Sham group, EM2-LI was significantly decreased in the spinal dorsal horn of Vincristine group (Fig. 2A). Using ELISA, we detected that compared to Naive group (80±5.1 ng/mg protein; 5d) and Sham group (83±4.2 ng/mg protein; 5d), spinal EM2 content was significantly decreased in Vincristine group at 5d (53±7.6 ng/mg protein). EM2 downregulation peaked at 2w (24±3.8 ng/mg protein) and thereafter persisted at low level (n = 10/group; *P*<0.05) (Fig. 2B). Thus, the time course change of spinal EM2 content was similar to that of mechanical allodynia. Furthermore, the content of EM2 was found to be significantly correlated to the paw withdrawal threshold in Vincristine group (P<0.001, r = 0.939) (Fig. 2C).

With regard to MOR expression in spinal cord, immunostaining (Fig. 2D) and Western blot (Fig. 2E) showed that there was no difference among Naive group, Sham group and Vincristine group at any time points tested. In all the rats, MOR expression was unchanged through the period tested.

**Figure 3:**
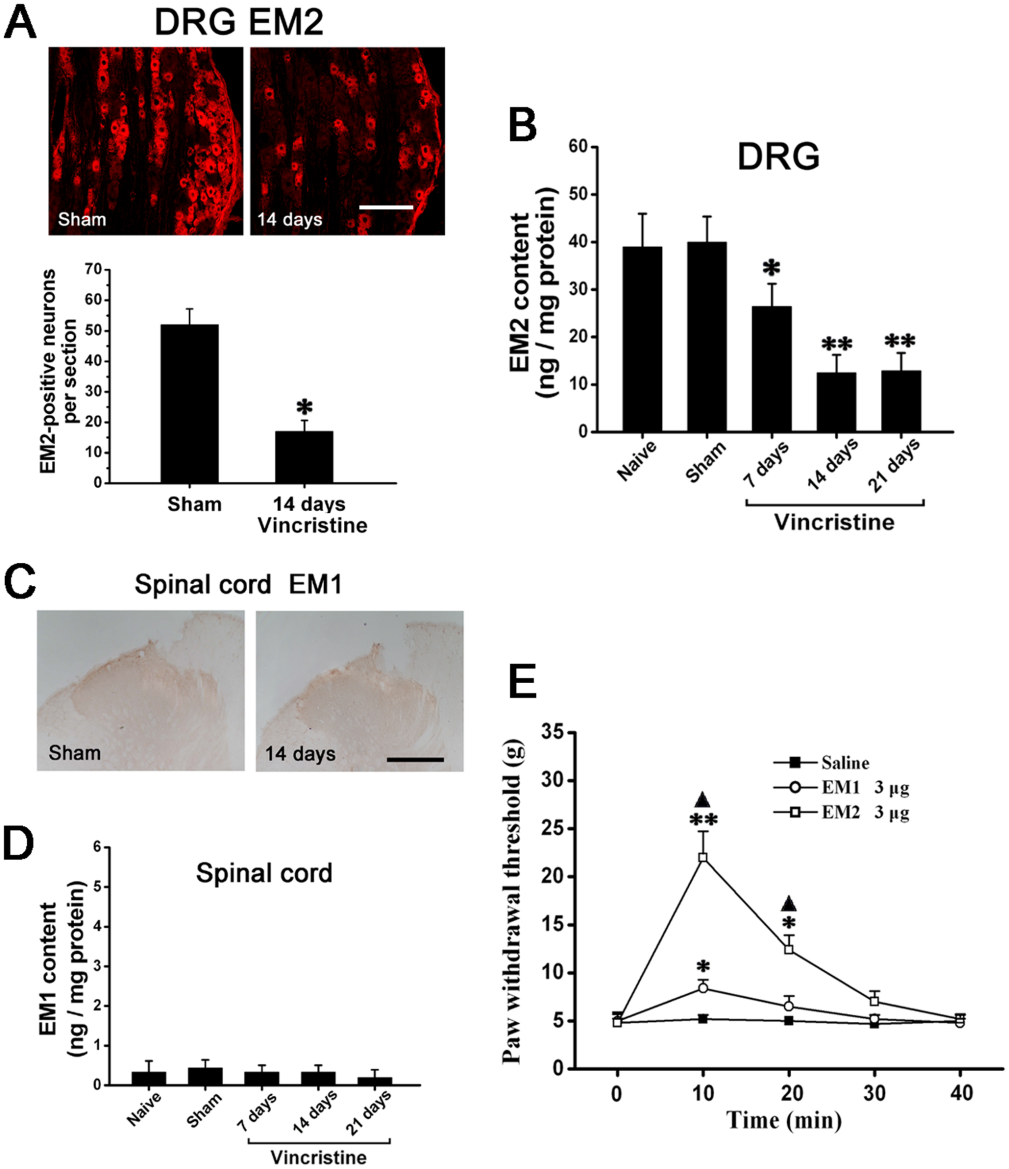
EM2 in dorsal root ganglion was significantly decreased, and there was almost no spinal EM1 in vincristine treated rats. (A) The number of EM2-immunopositive dorsal root ganglion neurons in Vincristine group was significantly decreased compared to Sham group. Bar = 100 µm. (B) Compared to Naive group and Sham group, ELISA showed that the EM2 content of dorsal root ganglion was significantly decreased in Vincristine group. (C) There was almost no EM1-immunopositive structures in spinal cord in Sham group and Vincristine group. (D) ELISA showed that the spinal cords of all the rats contained almost no EM1. (E) EM2 showed more effective antinociception than EM1. *P<0.05, **P<0.01 vs. Naive group and Sham group in (A) and (B). *P<0.05, **P<0.01 vs. Saline group; ▴ P<0.05 vs. EM1 group in (E).

Immunohistochemistry indicated that compared to Sham group, EM2-immunopositive neurons was significantly decreased in the dorsal root ganglion of Vincristine group (Fig. 3A). Using ELISA, we detected that compared to Naive group (39±7.1 ng/mg protein; 14d) and Sham group (41±5.9 ng/mg protein; 14d), the EM2 content of dorsal root ganglion was significantly decreased in Vincristine group (13±3.6 ng/mg protein; 14d) (Fig. 3B).

Immunohistochemistry indicated that there was no detectable EM1-immunopositive structures in spinal cord in the three groups (Fig. 3C). Using ELISA, we detected that the spinal cords of the three groups contained almost no EM1 (Fig. 3D). Furthermore, pain behavior test showed that EM2 exerted more effective antinociception compared with corresponding dose of EM1 (Fig. 3E).

### Decreased Spinal EM2 Contributed to Allodynia and Central Sensitization

**Figure 4:**
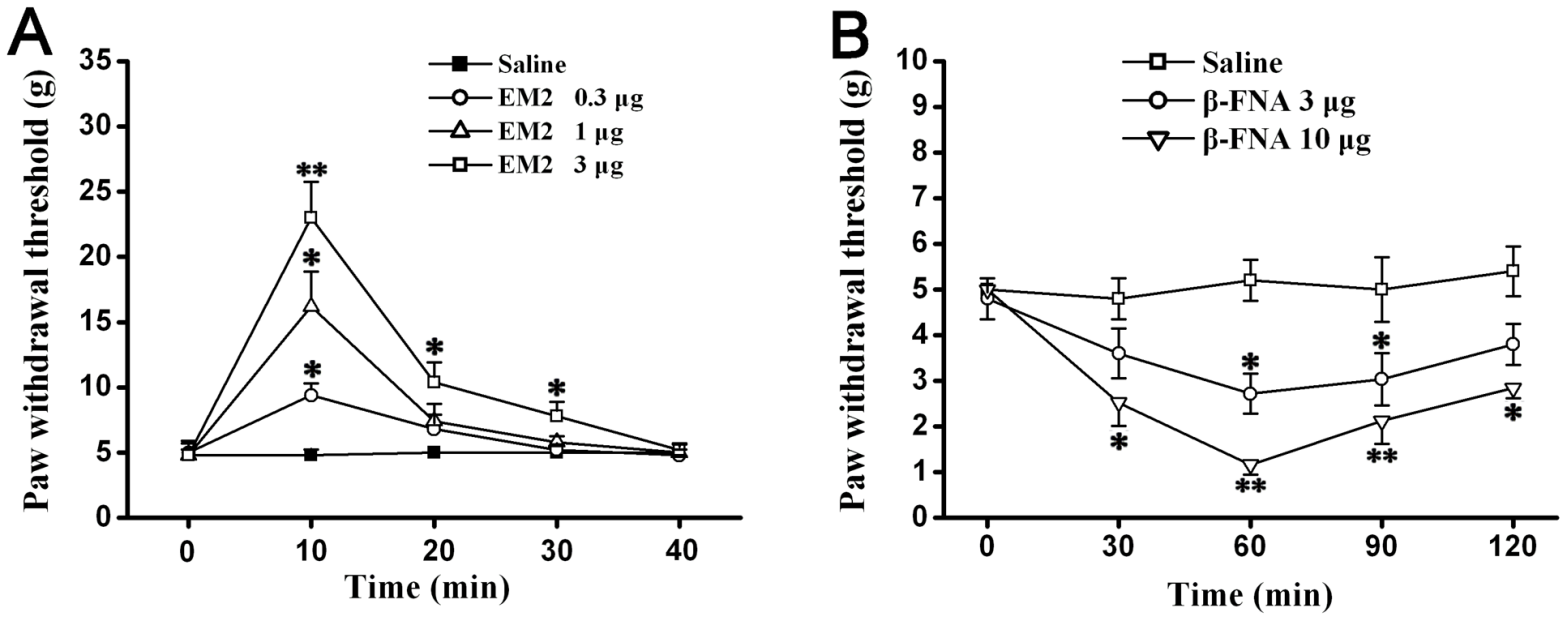
Decreased spinal EM2 contributed to mechanical allodynia in vincristine treated rats. (A) In Vincristine group, intrathecal injection of EM2 significantly attenuated the allodynia in a dose-dependent manner. (B) In Vincristine group, intrathecal administration of the mu-opioid receptor antagonist β-FNA made the mechanical allodynia more serious. *P<0.05, **P<0.01 vs. Saline group.

We injected EM2 or β-FNA intrathecally and observed their effects on mechanical allodynia in Vincristine group (2w). While the exogenous EM2 dose-dependently attenuated the allodynia (Fig. 4A), the mu-opioid receptor antagonist β-FNA facilitated the mechanical allodynia (Fig. 4B).

**Figure 5:**
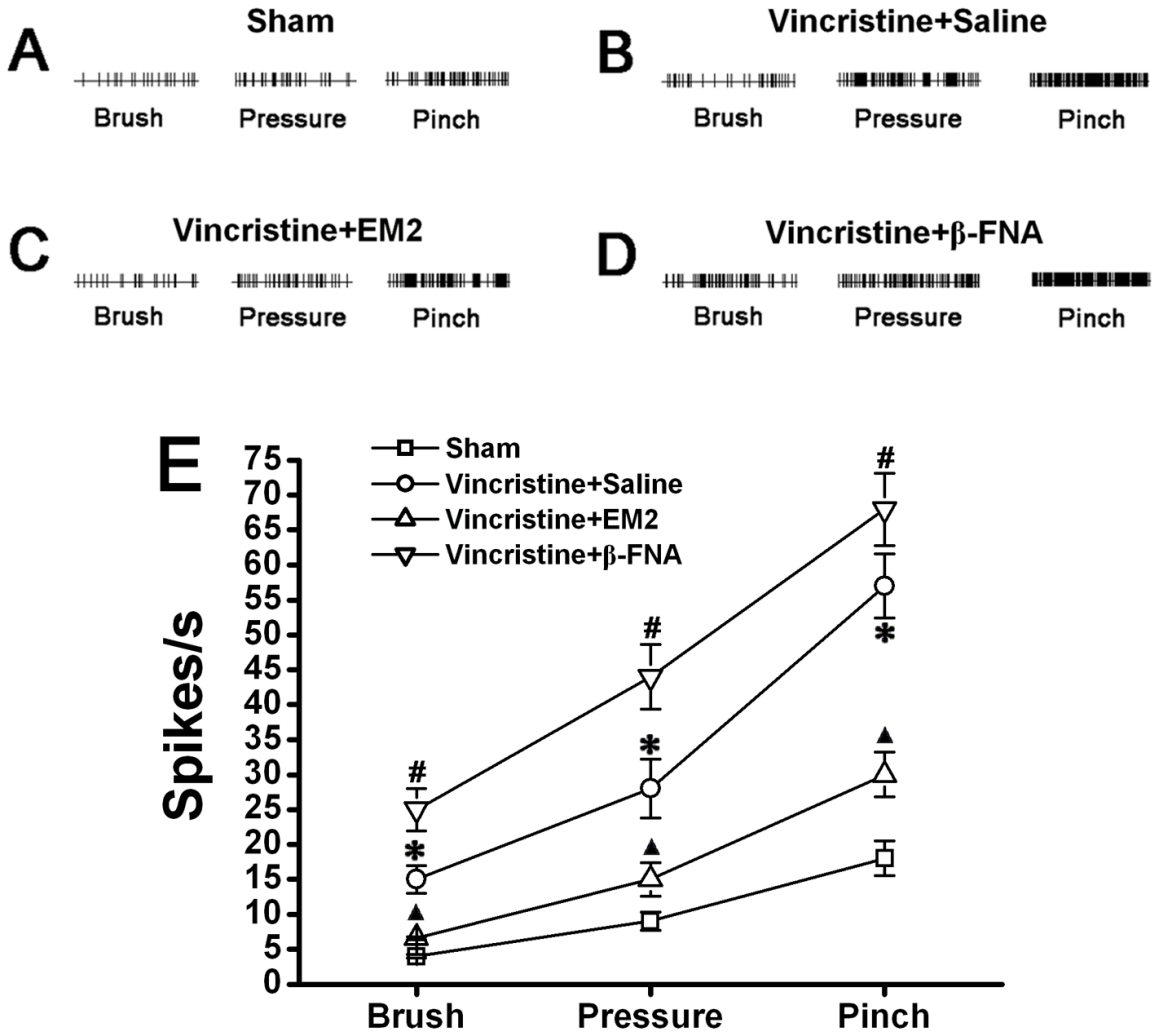
Decreased spinal EM2 contributed to spinal central sensitization in vincristine treated rats. Comparative recordings of responsiveness of spinal dorsal horn wide dynamic range (WDR) neurons to mechanical (brush, pressure and pinch) stimuli in Sham group (A), Vincristine +Saline group (B), Vincristine+EM2 group (C) and Vincristine+β-FNA group (D). (A–D) The responsiveness of WDR neurons was gradedly increased with the increase in mechanical intensity (brush, pressure and pinch). (E) Compared to Sham rats, the responsiveness of WDR neurons was significantly enhanced with a distinct leftward shift of the stimulus-response functional curve in vincristine treated rats. While the exogenous EM2 significantly attenuated the increased responsiveness, the mu-opioid receptor β-FNA aggratated the increased responsiveness. *P<0.05 vs. Sham group; ▴ or # P<0.05 vs. Vincristine+Saline group.

The responsiveness of WDR neurons was gradually increased with the increase in mechanical intensity (brush, pressure and pinch) (Fig. 5A–D). The stimulus-response functional curves for mechanical sensitivity of the spinal dorsal horn WDR neurons are shown in each group (Fig. 5E). Compared to Sham group, the responsiveness of WDR neurons was significantly enhanced with a distinct leftward shift of the stimulus-response functional curve in Vincristine group (2w), which indicated that spinal central sensitization occurred in Vincristine group rats (Fig. 5E). We injected EM2 or β-FNA intrathecally and observed their effects on increased responsiveness of WDR neurons in Vincristine group (2w). The increased responsiveness was significantly attenuated by EM2, whereas it was significantly facilitated by β-FNA (Fig. 5E).

### Spinal Decreased EM2 was Induced by Increased Activity of DPP IV, and Chemotherapy-induced Oxidative Stress might be the Causative Factor for Increased Activity of DPP IV

**Figure 6:**
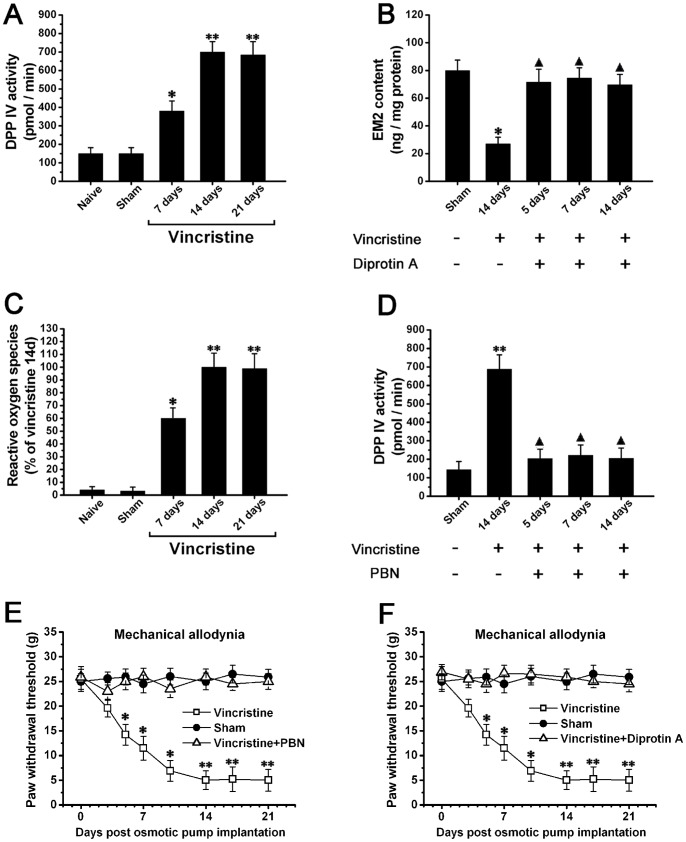
Spinal decreased EM2 was induced by oxidative stress mediated increased activity of dipeptidylpeptidase IV (DPP IV). (A) Compared to Naive group and Sham group, the activity of spinal DPP IV was significantly increased in Vincristine group. (B) A systemic treatment with diprotin A (inhibitor of DPP IV) blocked the decrement of spinal EM2 in Vincristine group. (C) Compared to Naive group and Sham group, the spinal reactive oxygen species was significantly increased in Vincristine group. (D) A systemic treatment with PBN (a scavenger for reactive oxygen species) significantly attenuated the increased activity of DPP IV in Vincristine group. (E) The treatment with PBN prevented the forming of mechanical allodynia in Vincristine group. (F) The treatment with diprotin A prevented the forming of mechanical allodynia in Vincristine group rats. *P<0.05, **P<0.01 vs. Naive group and Sham group; ▴ P<0.05 vs. Vincristine group at 14 days.

Compared to Naive group (150±20.2 pmol/min) and Sham group (145±19.3 pmol/min), the activity of DPP IV was significantly increased in Vincristine group (373±50.2 pmol/min) at 7d (P<0.05). The activity of DPP IV peaked at 2W (680±60.4 pmol/min) and persisted thereafter, which correlated with the changing course of spinal EM2 content (Fig. 6A). On the other hand, some Vincristine group rats were treated with diprotin A (inhibitor of dipeptidylpeptidase IV), which produced a significant inhibition of the decrement of spinal EM2 (Fig. 6B). These data indicated that increased activity of DPP IV contributed to decreased spinal EM2 after chemotherapy.

We observed that spinal reactive oxygen species was significantly increased in Vincristine group (Fig. 6C), and a systemic treatment with PBN (reactive oxygen species scavenger) significantly reduced the increased activity of DPP IV (Fig. 6D), which indicated that Vincristine-induced oxidative stress may mediate the development of increased activity of DPP IV in Vincristine group.

In addition, the treatment with PBN and diprotin A prevented the occurrence of mechanical allodynia in Vincristine group rats (Fig. 6E and F). On the other hand, neither PBN nor diprotin A treatment affected the motor performance of Vincristine group rats, the motor function of all the rats maintained at normal level from 1d to 28d (data not shown).

## Discussion

The mechanisms underlying CNP are unclear and need to be investigated further [28]. To our knowledge, there are no analgesic drugs that could be labeled both safe and effective in treating CNP [20], [34]–[38]. To identify such therapeutic strategies, it is necessary and crucial to elucidate the molecular mechanisms resulting in CNP following chemotherapy. This study is the first to provide conclusive evidence that decreased levels of EM2 in the spinal cord contribute to allodynia and central sensitization in CNP. We also show that this decrease is likely due to increased activity of dipeptidylpeptidase IV (DPP IV), caused by chemotherapy-induced oxidative stress.

### Technical Considerations

In this study, we modeled CNP in rats by implanting a mini-osmotic pump to provide continuous infusion of vincristine for 2 weeks, such that its concentration in blood is maintained at a consistent level [19], [20]. Compared to other previously established methods such as consecutive intraperitoneal injections, our method has several advantages: the procedure needs to be performed only once, is quick, ensures reliable intravenous drug delivery, and animals show good tolerance [19].

Although endogenously expressed endomorphins have been isolated from tissue, the identity of the gene encoding the precursor protein from which the endomorphin peptides are derived has not been identified. Due to the small molecular weight of EMs (<1 kDa), PCR and Western blotting methods could not be used to perform quantitative analyses. However, researchers have successfully used ELISA to precisely determine the content of spinal EM2 [26].

### Decreased Spinal EM2 Expression after Chemotherapy Contributes to Allodynia in CNP Rat

CNP is characterized by allodynia, hyperalgesia and spontaneous pain, features that are also found in other forms of neuropathic pain. Although it is still unclear how chemotherapeutics interact with the nervous system to induce changes in pain sensation and behavior, the neural mechanisms underlying CNP may be similar to other forms of neuropathic pain [4]. Like neuropathic pain (and inflammatory pain) induced by trauma and diabetes, hypersensitivity of C-fiber nociceptors following treatment with the anti-tumor agent vincristine, has been noted in some neurophysiological studies [39]. Also reported is central sensitization of the wide dynamic range neurons of the spinal cord in response to vincristine treatment [40]. Studies investigating the mechanisms causing CNP have hypothesized that the fundamental pathology of CNP is a toxic effect on axonal mitochondria. The subsequent impairment in mitochondrial function may lead to membrane depolarization and action potentials [41]. More research is needed to corroborate this hypothesis and further dissect the pathways contributing to CNP.

It has been shown that after partial ligation of the sciatic nerve, EM2-LI is significantly decreased in the spinal dorsal horn ipsilateral to the nerve injury based on immunohistochemical analysis. Smith et al first reported the decrease in endogenous EM2 levels in chronic pain [14]. We made similar findings in this study, and showed, using immunohistochemistry and ELISA, a significant reduction in EM2 in the spinal dorsal horn and dorsal root ganglion in a rat CNP rat model. Since measurement of the concentration of neuropeptides by immunohistochemistry is only semiquantitative, we used ELISA to determine more accurately the change in spinal EM2 levels.

Changes in the opioidergic systems have been studied in other neuropathic pain models. Several reports have shown an upregulation of dynorphin using immunocytochemistry [42], radioimmunoassay [43] and mRNA [44] measurements after nerve injury. Although the predominant cellular effect of opioids is inhibition of neuronal excitability, dynorphin has been shown to enhance dorsal horn neuronal hyperexcitability in nerve injury models of chronic pain [43]. Administration of dynorphin can induce allodynia that in turn can be attenuated by endomorphins [45]. Metenkephalin, another naturally occurring opioid peptide, has been shown not to change significantly after nerve injury [44], [46]. Our results provide the first evidence that reduction in the levels of an endogenous opioid in primary afferents is significantly associated with CNP. This indicates that the chronic pain associated with CNP might be due to the loss of an inhibitory effect on pain signal transmission.

Examination of several pain models revealed significant alterations in the expression of MOR in the spinal dorsal horn and dorsal root ganglion. For example, MOR expression is decreased in the spinal dorsal horn and the inhibitory effect of MOR agonists is reduced in neuropathic pain and bone cancer pain [47], [48]. Peripheral inflammation increases MOR expression in the dorsal horn and dorsal root ganglion [49], [50], with an increase in the inhibitory effect of MOR agonists [51]. Interestingly, this study found that MOR expression in the spinal cord remains unchanged after vincristine treatment. It therefore seems that the reduction in spinal EM2 level does not induce a compensatory increase in MOR expression, suggesting that spinal MOR may not be involved in the initiation and maintenance of chronic pain in CNP rats.

Although Smith et al provided the first evidence of a decrease in endogenous EM2 levels in the spinal cord following partial ligation of the sciatic nerve, they did not perform behavioral tests to assess pain and to evaluate the effect of intrathecal administration of exogenous EM2 or the mu-opioid receptor antagonist β-FNA [14]. Therefore, the causal relationship between decreased spinal EM2 and pain behavior could not be established. In this study, we demonstrated that the mechanical allodynia in CNP rats is attenuated by intrathecal administration of EM2, while administration of β-FNA further decreased the von Frey threshold for mechanical sensitivity. These behavioral results further elucidated the contribution of decreased spinal EM2 levels to decreased endogenous inhibitory influence on pain transmission. The loss of endogenous pain inhibition may be one of the most important mechanisms underlying the chronic pain associated with CNP.

EM1 and EM2 are structurally different by only one amino acid residue; hence, some comparative studies were performed to reveal the functional difference between EM1 and EM2. EM1-immunopositive structures are predominantly located in the brain while EM2-immunopositive structures are mainly found in the lower brainstem and spinal cord. While EM2-immunopositive structures exist at high density in the spinal dorsal horn, almost no EM1-immunopositive structures could be detected at the spinal level [52], [53]. Similar to previous reports, our immunohistochemistry and ELISA assays showed there is almost no expression of EM1 in the spinal cord of both control and vincristine-treated rats. Although there is enough evidence indicating both EM1 and EM2 play pivotal roles in inhibiting nociceptive transmission at the spinal cord level, it has also been shown that EM2 is a more potent analgesic in the spinal cord than EM1 when administered intrathecally [54], [55]. Similarly, our pain behavior test showed that EM2 has a more effective antinociceptive function than similar doses of EM1. An electrophysiological study suggested that the difference in behaviorally examined antinociceptive functions of EM1 and EM2 may be due to differences in their enzymatic degradation [56].

### The Decrease in Spinal EM2 Levels Contribute to Central Sensitization of Spinal Neurons in CNP Rats

Emerging reports have suggested that EM2 expression in the spinal dorsal horn might functionally affect nociception by binding to pre-synaptically localized mu-opioid receptors causing inhibition of neurotransmitter release, and by binding to post-synaptic mu-opioid receptors causing hyperpolarization of spinal dorsal horn neurons [9], [10]. Therefore, a reduction in EM2 in the spinal cord could lead to a loss of inhibition and may contribute to central sensitization. Spinal dorsal horn wide dynamic range (WDR) neurons are more plastic following tissue injury and are believed to be responsible for the spinally-organized central sensitization [57]. In this study, we found that WDR neurons show significantly increased responsiveness to stimuli during CNP. This suggests that spinal central sensitization does occur in CNP rats. Our findings corroborate previous reports of central sensitization following vincristine treatment [40]. Most importantly, intrathecal administration of exogenous EM2 significantly attenuated the central sensitization, and administration of β-FNA exerted opposite effects. Our findings demonstrate that the decrease in spinal EM2 plays an important role in causing the excessively increased activity of dorsal horn neurons.

### Decreased Spinal EM2 Expression is Caused by Increased Activity of DPP IV

DPP IV is a membrane-bound serine proteinase that removes dipeptides from the amino terminus of peptides containing proline as the penultimate amino acid [58]. Endomorphins satisfy this substrate requirement, and were shown to be excellent substrates for DPP IV, which can liberate the N-terminal Tyr-Pro with high activity [59]. The use of DPP IV inhibitor enhanced and prolonged EM2-induced analgesia, and established the function of this enzyme in endomorphin degradation [60]. In the current study, we detected a significant increase in the activity of spinal DPP IV following vincristine treatment. We therefore hypothesize that the decrease in spinal EM2 levels is caused by chemotherapy-induced increase in the activity of DPP IV. We confirmed this by demonstrating that systemic treatment with diprotin A (an inhibitor of DPP IV) can block the downregulation of spinal EM2.

### Chemotherapy-induced Oxidative Stress Might Cause Increased DPP IV Activity

Primary afferent sensory terminals and spinal dorsal horn are regions of high metabolic demand. The terminals of sensory afferents contain an unusually high concentration of mitochondria [61], [62]. It has been shown that chemotherapy-evoked neuropathy is caused by a toxic effect on axonal mitochondria [41]. Impaired mitochondrial function in turn produces an excessive amount of reactive oxygen species (ROS), which induces significant oxidative stress [63]. In this study, we examined the level of reactive oxygen species in the spinal cord of CNP rats, and found them to be significantly increased following vincristine treatment.

Oxidative stress has been shown to influence the activity of DPP IV [24]. Based on our observations, we hypothesized that chemotherapy-induced oxidative stress may be a key mechanism causing increased activity of DPP IV. We confirmed this by treating CNP rats with PBN (a scavenger of reactive oxygen species) and showing a significant reduction in the increased activity of DPP IV. Hence, oxidative stress seems to induce the increase in DPP IV activity in vincristine treated rats.

In summary, our study of a rat CNP model provides the first evidence that spinal EM2 levels, but not MOR, are significantly decreased in CNP. We have shown that the allodynia and central sensitization associated with CNP is induced by a loss of endogenous inhibitory influence on pain transmission. Finally, our data suggest that chemotherapy-induced oxidative stress induces an increase in the activity of DPP IV, which in turn contributes to the reduction in spinal EM2 expression.
